# Therapy of cutaneous leishmaniasis caused by *Leishmania braziliensis* with fluconazole

**DOI:** 10.1111/dth.15060

**Published:** 2021-07-18

**Authors:** Stefano Veraldi, Maurizio Romagnuolo, Marco Cusini, Carlo Alberto Maronese

**Affiliations:** ^1^ Department of Pathophysiology and Transplantation Università degli Studi, IRCCS Foundation Ca' Granda Ospedale Maggiore Policlinico Milan Italy

Dear Editor,

Literature data on the use of fluconazole in cutaneous leishmaniasis (CL) by *Leishmania braziliensis* are poor and conflicting.^1^, ^2^, ^3^, ^4^

We present four patients with severe CL by *L. braziliensis* who were successfully treated with fluconazole (Supplementary Table 1).

The patients were four Caucasians who acquired CL after trips to Brazil, Bolivia, and Colombia. All patients were previously, but unsuccessfully, treated with different drugs. All patients were subjected to general and dermatologic examination, laboratory tests, cytologic, and histopathologic examinations, culture in Novy‐MacNeal‐Nicolle medium and polymerase chain reaction (PCR).

In all patients CL was characterized by ulcerative lesions (Figure 1A). Laboratory tests were within normal ranges. Cytologic and histopathologic examinations revealed the presence of *Leishmania* spp. Culture was positive for *Leishmania* spp. PCR was positive for *L. braziliensis*. Leishmanin Skin/Montenegro test was carried out in three patients, who resulted to be positive.

**FIGURE 1:**
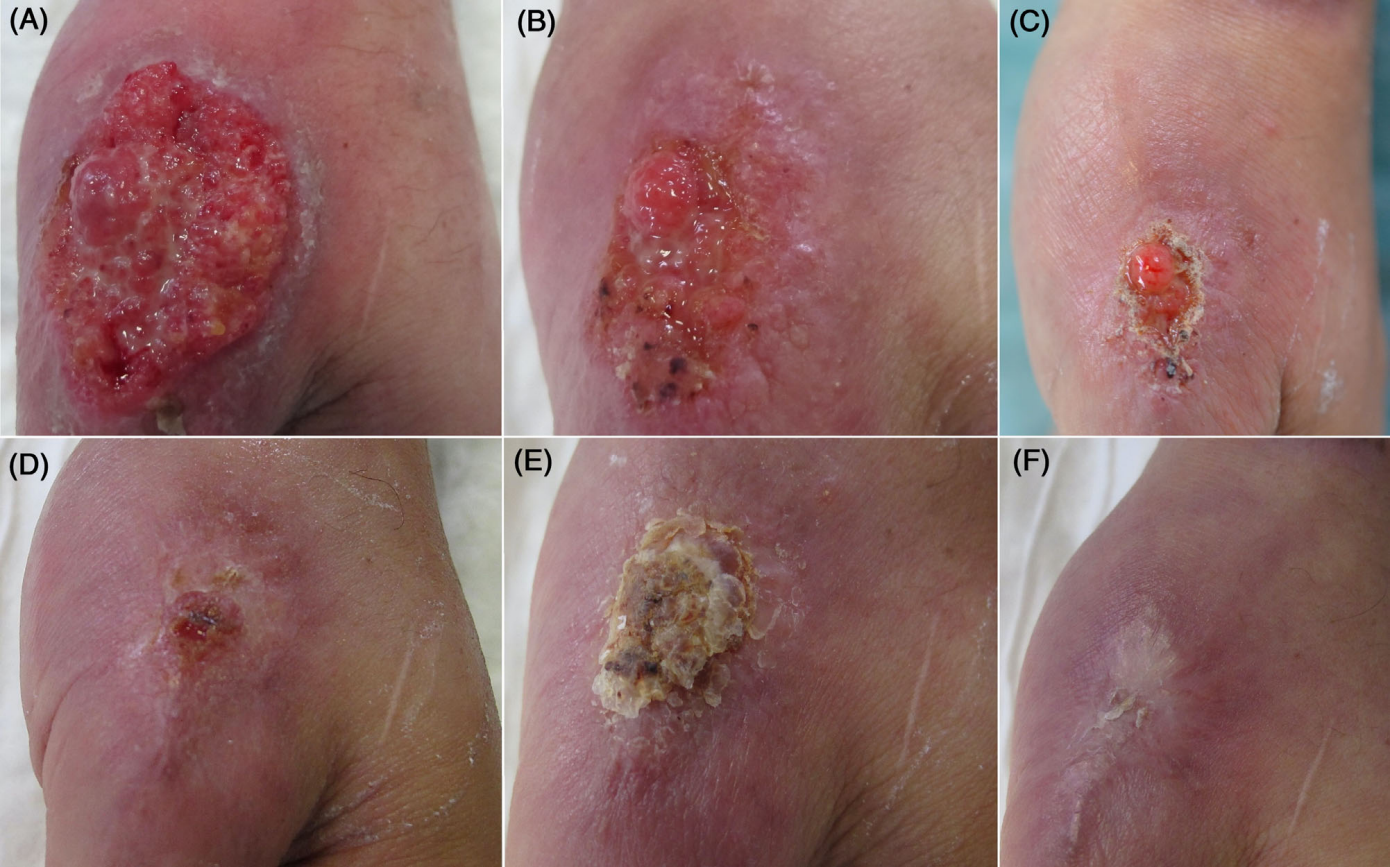
(A–F) Patient ≠1. Evolution of the clinical picture of CL from the beginning of the therapy with fluconazole (A) to the healing (F), 11 months later. CL, cutaneous leishmaniasis

Fluconazole was used at the dosage of 400 mg/day for 6–11 months. All patients were examined every month. Laboratory examinations were performed every 2 months.

The first clinical improvement was observed 4–6 weeks after the beginning of the therapy. In all patients, complete remission was obtained (Figure 1A–F). Follow‐up (≤7 years and 11 months) was negative in all patients. Side effects were local itching, weakness, arthralgia, myalgia and gastralgia (one patient), and local pain and weakness (one patient). In one patient mild increase in azotaemia was detected. In all patients it was unnecessary to stop the therapy.

Azole and triazole drugs inhibit the growth of *Leishmania* spp. by inhibiting the cytochrome P‐450–mediated 14α‐demethylation of lanosterol, blocking ergosterol synthesis, and causing accumulation of 14α‐methyl sterols.^5^

A recent study demonstrated that *L. braziliensis* is sensitive in vitro to fluconazole.^6^ The first report on the use of fluconazole in CL by *L. braziliensis* was published in 2007: no clinical results were observed in three Austrian soldiers who acquired the infection in Belize.^1^ Sousa et al.^2^ successfully used fluconazole in 28 patients. They started with a dosage of 5 mg^−1^kg^−1^day^−1^ and reached 8 mg^−1^kg^−1^day^−1^. The duration of the therapy was 4–12 weeks. With the low dosage, 75% patients recovered; with 8 mg^−1^kg^−1^day^−1^, the cure rate was 100%. The drug was well tolerated. A randomized, controlled study was carried out to compare the efficacy and safety of fluconazole (6.5–8 mg^−1^kg^−1^day^−1^ for 28 days) versus pentavalent antimony (20 mg^−1^kg^−1^day^−1^ for 20 days). A total of 53 patients were included: 27 patients were treated with fluconazole and 26 with antimony. Initial cure rates (two months after treatment) were of 22.2% (6/27 patients) in the fluconazole group and 53.8% (14/26 patients) in the antimony group. No relapses were observed. The frequency of adverse events in the antimony and fluconazole groups was similar: 34.6% versus 37%, respectively. In the group of patients treated with fluconazole, dizziness was observed in 22.2% of them, nausea in 11.1% and headache in 7.4%. One patient discontinued the treatment because of headache and dizziness. According to this study, fluconazole was not considered as an effective therapy.^4^

Our experience on the use of fluconazole in CL is based on approximately 30 patients: we successfully treated patients with CL by *L. panamensis* ^7^ and *L. infantum*.^8^, ^9^ Fluconazole was also successfully used in pediatric patients with CL caused by *L. major* and *L. tropica*. Furthermore, no important side effects were observed.^10^, ^11^

It is possible that the duration of the therapy is important. As previously mentioned, we used fluconazole for 6–11 months: in the first 4–6 weeks of therapy clinical results were slow and partial. In the previously cited study,^4^ fluconazole was used for only 28 days. We also observed that a long therapy induced neither important side effects nor laboratory abnormalities.

In spite of the small number of patients treated, we think that fluconazole can be taken into consideration in patients with CL by *L. braziliensis* in those countries where pentavalent antimony is no more marketed.

## CONFLICT OF INTEREST

The authors declare no potential conflict of interest.

## Supporting information


**Supplementary Table 1** Patients' characteristics.

## Data Availability

The data that support the findings of this study are available from the corresponding author upon reasonable request.
