# Supplementary material for: Therapy of cutaneous leishmaniasis caused by Leishmania braziliensis with fluconazole
Source: Dermatol Ther. 2021 Jul 18;34(5):e15060. doi: 10.1111/dth.15060 (PMC8596449; doi:10.1111/dth.15060)
Supplement: Supplementary file 1 — Supplementary Table 1 Patients' characteristics. [file DTH-34-e15060-s001.docx]

**Supplementary Table 1**. Patients’ characteristics.

| **Patients/Gender/Age (years)** | **Country of infection** | **Location of CL** | **Symptoms and signs** | **Previous therapies** | **Duration of fluconazole therapy** | **Side effects/ Laboratory abnormalities** |
| --- | --- | --- | --- | --- | --- | --- |
| 1/male/35 | Brazil and Bolivia | Left hand and forearm | Lymphangitis; arterial hypertension; electrocardiographic abnormalities | Gentamicin; ciprofloxacin | 10 months | Mild increase in azotaemia |
| 2/male/58 | Brazil | Right ear | Weakness | Paromomycin | 9 months | Itching; weakness; arthralgia; myalgia; gastralgia |
| 3/male/50 | Brazil and Bolivia | Right hand | - | Paromomycin | 6 months | - |
| 4/male/26 | Brazil and Colombia | Right ear | Pain; weakness | Amoxicillin; levofloxacin; liposomal amphotericin B | 11 months | Pain; weakness |
